# Hyponatremia induced Brugada syndrome mimicking ST segment elevation myocardial infarction

**DOI:** 10.1002/joa3.12617

**Published:** 2021-08-14

**Authors:** Pattara Rattanawong, Vichai Senthong

**Affiliations:** ^1^ Department of Cardiovascular Medicine Mayo Clinic Phoenix AZ USA; ^2^ Cardiovascular Unit Faculty of Medicine Khon Kaen University Khon Kaen Thailand

**Keywords:** Brugada syndrome, hyponatremia

## Abstract

Seventy‐three year‐old male with history of diabetes, hypertension, and chronic kidney disease stage 3 presented with epigastric pain and hyponatremia. ECG showed new ST segment elevation at precordial leads consistent with Cove‐type Brugada ECG pattern. Cardiac catheterization revealed non‐significant coronary artery stenosis. He experienced pre‐syncope and palpitations a year prior to admission with family history sudden cardiac death. Brugada syndrome was diagnosed. Cove‐type Brugada ECG pattern and palpitations resolved with corrected sodium to 135.

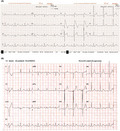

Brugada syndrome is an autosomal dominant inherited disease with variable penetrance mutations that was described by sodium channels dysfunction. Men are at risk eight to nine times than woman to express the phenotype of mutation that leads to develop Brugada syndrome.^1^ Brugada syndrome is reported to be the most common cause of natural death in men younger than 50 years old. The clinical manifestation of Brugada syndrome include ventricular fibrillation (VF) or aborted SCD, syncope, nocturnal agonal respiration, palpitations or chest discomfort. The symptoms usually present in the fourth decade of life and the mean age of sudden death is 41 ± 15 years old.^1^ A Brugada pattern can be unmasked by various conditions including hypokalemia, hyperkalemia, hypercalcemia, certain drugs, and fever.^1^, ^2^ Previous case reports presented asymptomatic type‐1 Brugada pattern unmasked by hyponatremia and resolved with sodium correction.^3^, ^4^, ^5^ Here, we present another case of the Brugada syndrome being unmasked by hyponatremia.

A 73‐year‐old male with a history of diabetes, hypertension, and stage 3 chronic kidney disease experienced epigastric pain associated with nausea and vomiting, shortness of breath, and palpitations for 6 hours before presenting to the hospital. His electrocardiogram showed new ST segment elevation at the precordial leads (Figure 1A). He was referred for emergency cardiac catheterization. On arrival, he was afebrile with blood pressure 140/72 mmHg, heart rate 97 bpm, and respiratory rate 20 bpm. Further examination revealed a flat neck vein and poor skin turgor. The lab results were remarkable for high‐sensitivity cardiac troponin T (hs‐cTnT) level of 20.41 ng/L, which did not increase after six hours (21.2 ng/L), a white blood cell count of 3.5 cells/μL, sodium levels of 126 mEq/L, potassium levels of 4.7 mEq/L, creatinine levels of 1.3 mg/dL, calcium levels of 7.8 mg/dL, and magnesium levels of 2.2 mg/dL. He was taking aspirin 81 mg daily, simvastatin 10 mg daily, gabapentin 300 mg twice a day, and insulin injection. The patient's chest X‐ray was unremarkable. An echocardiogram showed a normal ejection fraction of 65% without significant valvular lesions. Cardiac catheterization revealed non‐significant coronary artery stenosis (40% stenosis of the proximal left anterior descending artery, 30% stenosis of the proximal left circumflex artery, and 40% stenosis of the middle‐segment of right coronary artery) without evidence of coronary artery spasm including conus branch. A coved‐type Brugada electrocardiogram pattern was then considered (Figure 1A). The patient had experienced one episode of pre‐syncope and palpitations a year prior to admission with a family history of sudden cardiac death in three males who were second‐degree relatives. However, genetic test was not done because of limited resources. The coved‐type Brugada electrocardiogram pattern persisted during cardiac catheterization and resolved within 10 hours with corrected sodium to 135 mEq/L (Figure 1B) as well as all symptoms (epigastric pain associated with nausea, vomiting, shortness of breath, and palpitation). The Brugada electrocardiogram pattern was unmasked by the presence of hyponatremia and the patient was diagnosed with Brugada syndrome. Given no significant coronary artery lesion, troponin elevation in this case was likely because of false positive from CKD. Implantable cardioverter defibrillation was recommended; however, patient refused. There was no arrhythmic events and ECG showed normal sinus rhythm without Brugada pattern at 4 years follow‐up.

**FIGURE 1:**
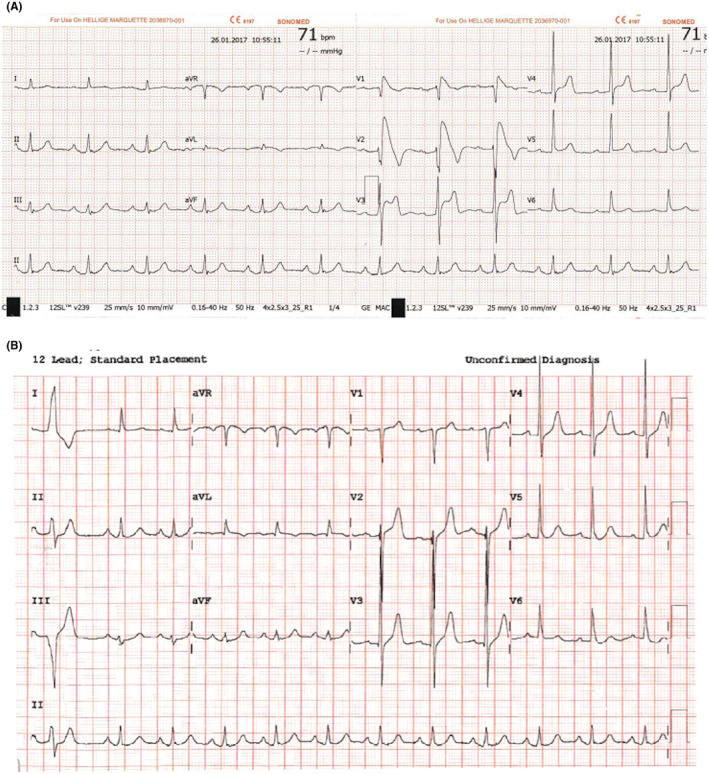
A, ST segment elevation at precordial leads consistent with a coved‐type Brugada pattern with hyponatremia (serum sodium 126 mEq/L). B, The first ECG which documented resolved coved‐type ST segment elevation at precordial leads with normal serum sodium of 135 mEq/L

## 1 DISCUSSION

Diagnosis of Brugada syndrome is challenging in cases of acute epigastric pain and palpitations with new ST segment elevation. The cause of epigastric pain could be an atypical chest pain from Brugada syndrome versus excessive nausea and vomit. Differential diagnosis of Brugada syndrome mimicking ECG includes ST elevation myocardial infarction, ventricular aneurysm, and early repolarization. An echocardiogram and coronary angiogram are essential in order to rule out structural and ischemic etiologies. A family history of sudden cardiac death is also useful for diagnosis but not for risk stratification. A Brugada pattern electrocardiogram can be unmasked by the presence of various conditions including hypokalemia, hyperkalemia, hypercalcemia, certain drugs, and fever.^1^, ^2^ Previous reports have demonstrated that a Brugada electrocardiogram pattern can be induced by hyponatremia.^3^, ^4^, ^5^ The mechanism is unclear. Hyponatremia decreases the Na^+^ inward current, *V*_max_, and conduction velocity. Hyponatremia also decreases effective refractory period shortening in the epicardium but not in the endocardium.^6^ Given the difference in the magnitude of regional effective refractory period shortening and the reversible Brugada pattern after normalization of sodium level, an alteration of trans‐membrane sodium gradient could be the explanation mechanism.

## 2 LEARNING POINTS

This case report represents Brugada syndrome being unmasked by hyponatremia. Brugada syndrome should be considered in transient ST elevation at precordial leads without significant coronary artery disease or structural heart abnormality.

## DISCLOSURES

The authors declare no conflict of interests for this article. This study was approved by the Khon Kaen University Ethics Committee for Human Research (HE601200) and was conducted in accordance with the Declaration of Helsinki.

## References

[1] Priori SG, Wilde AA, Horie M, Cho Y, Behr ER, Berul C, et al. HRS/EHRA/APHRS expert consensus statement on the diagnosis and management of patients with inherited primary arrhythmia syndromes: document endorsed by HRS, EHRA, and APHRS in May 2013 and by ACCF, AHA, PACES, and AEPC in June 2013. Heart rhythm. 2013;10(12):1932–63. 24011539 10.1016/j.hrthm.2013.05.014

[2] Rattanawong P, Vutthikraivit W, Charoensri A, Jongraksak T, Prombandankul A, Kanjanahattakij N, et al. Fever‐induced brugada syndrome is more common than previously suspected: a cross‐sectional study from an endemic area. Ann Noninv Electrocardiol. 2016;21(2):136–41. 10.1111/anec.12288 PMC6931454 26178440

[3] Hunuk A, Hunuk B, Kusken O, Onur OE. Brugada phenocopy induced by electrolyte disorder: a transient electrocardiographic sign. Ann Noninv Electrocardiol. 2016;21(4):429–32. 10.1111/anec.12350 PMC6931746 26910573

[4] Tamene A, Sattiraju S, Wang K, Benditt DG. Brugada‐like electrocardiography pattern induced by severe hyponatraemia. Europace. 2010;12(6):905–7. 20185483 10.1093/europace/euq034

[5] Alvarez PA, Vázquez Blanco M, Lerman J. Brugada type 1 electrocardiographic pattern induced by severe hyponatremia. Cardiology. 2011;118(2):97–100. 21540589 10.1159/000327089

[6] Wolk R, Kane KA, Cobbe SM, Hicks MN. Regional electrophysiological effects of hypokalaemia, hypomagnesaemia and hyponatraemia in isolated rabbit hearts in normal and ischaemic conditions. Cardiovasc Res. 1998;40(3):492–501. 10070489 10.1016/s0008-6363(98)00200-4

